# Gastrointestinal stromal tumors of the small intestine: the challenge of diagnosis and the outcome of management

**DOI:** 10.1186/s12957-023-02968-0

**Published:** 2023-03-09

**Authors:** Hosam Hamed, Mohamed Abdel Wahab, Youssif Elmahdy, Rihame M. Abd El-Wahab, El-Sayed Abou El-Magd

**Affiliations:** Department of General Surgery, Faculty of Medicine, Gastrointestinal Surgical Center GISC, Mansoura University, Gehan Street, Al Dakahlia Governorate 35511 Mansoura, Egypt

**Keywords:** Small intestine, Gastrointestinal stromal tumors, Anemia of unknown origin

## Abstract

**Purposes:**

Gastrointestinal stromal tumor (GIST) is a rare small intestinal tumor. Most patients usually report long-period complaints due to difficult diagnoses. A high grade of suspicion is required for early diagnosis and initiation of the proper management.

**Methods:**

A retrospective study of all patients with small intestinal GIST who were operated in the period between January 2008 and May 2021 at Mansoura University Gastrointestinal Surgical Center (GIST).

**Results:**

Thirty-four patients were included in the study with a mean age of 58.15 years (± 12.65) with a male to female ratio of 1.3:1. The mean duration between onset of symptoms and diagnosis was 4.62 years (± 2.34). Diagnosis of a small intestinal lesion was accomplished through abdominal computed tomography (CT) in 19 patients (55.9%). The mean size of the tumor was 8.76 cm (± 7.76) ranging from 1.5 to 35 cm. The lesion was of ileal origin in 20 cases (58.8%) and jejunal in 14 cases (41.2%). During the scheduled follow-up period, tumor recurrence occurred in one patient (2.9%). No mortality was encountered.

**Conclusion:**

Diagnosis of a small bowel GISTs requires a high grade of suspicion. Implementing new diagnostic techniques like angiography, capsule endoscopy, and enteroscopy should be encouraged when suspecting these lesions. Surgical resection is always associated with an excellent postoperative recovery profile and very low recurrence rates.

## Introduction

Gastrointestinal stromal tumors (GISTs) are the commonest mesenchymal tumor of the gastrointestinal tract [1]. However, it represents only about 1 – 2% of total primary gastrointestinal malignancies [2–4]. This tumor could occur at any portion of the gastrointestinal tract. The stomach is the commonest affected region (40 – 60%), followed by the small bowel (25 – 30%) [5–7].

GISTs arises from, Reviewer (2) the interstitial cell of Cajal [8]. Most of these tumors have a characteristic mutation in KIT (oncogenic mutations of the *KIT* receptor tyrosine kinase gene) Reviewer (2) or platelet-derived growth factor receptor alpha (PDGFRA). Conversely, succinate dehydrogenase deficiency Reviewer (2) is less frequent [9–11]. These tumors could present with different manifestations, including luminal GI bleeding, pain, mass, or incidentally Reviewer (2) discovered when performing radiological examination for other indications [12–14]. Of note, about 10 – 20% of these patients present with metastatic disease [15].

The diagnosis of small bowel GIST is considered challenging for many surgeons and physicians [16, 17]. Multiple factors contribute to this phenomenon. First of all, the incidence of GISTs is low in general, and its presenting symptoms are non-specific. In addition, radiological assessment may prove difficult due to the wide variation of radiological appearances. Reviewer (2) Also, the presence of surrounding bowel loops may overlap the tumor [18].

Finally, the small intestine area is difficult to assess via conventional endoscopies like upper and lower GI endoscopies. Although the small bowel could be visualized by enteroscopy or capsule endoscopy, these facilities are not present in multiple surgical centers due to high financial cost [19–22]. Consequently, patients with such pathology are often misdiagnosed and usually express a long duration of symptoms [16, 17].

The current study aims to discuss the presentation, diagnosis, surgical management, and clinical outcomes of patients diagnosed with small bowel GIST.

### Patients and methods

This is a retrospective cohort study of all patients who were diagnosed with small intestinal GIST and were operated on at Mansoura University Gastrointestinal Surgical Center (GISC) between January 2008 and May 2021. The study was approved by the local ethical committee and Institutional Review Board of Mansoura University, IRB code (R.22.10.1909.R1.R2).

Patient data were retrieved from an internal web-based registry system. The study included cases diagnosed with small intestinal GIST and classified as class I, II and III according to the American Society of Anesthesiologists (ASA) [23]. Exclusion criteria were cases with metastatic disease or who had ASA class more than III. Reviewer (2).

The radiological assessment included abdominal ultrasonography and triphasic pelviabdominal computed tomography (CT), while angiography was done for selected cases. The endoscopic assessment was performed by upper and/or lower endoscopy according to the patient complaint, tumor location, and relation to the remaining organs.

All operations were performed under general anaesthesia. Reviewer (2) After abdominal exploration, the lesion site, together with its relation with surrounding organs, was evaluated. The involved bowel part was exteriorized, and the lesion was resected with a sufficient gross safety margin. If there was an attachment to the surrounding organs, en-bloc resection was performed to avoid rupture of the mass.

Oral fluids were often started on the 2nd or 3rd postoperative day. Patients were discharged after full intake without complications. Postoperative morbidity and mortality were noted and recorded.

We scheduled follow-up visits for these cases at 1, 2, and four weeks after the operation, then every three months during the initial three years. These visits were rescheduled every six months till the end of the 5th year. During these visits, a clinical assessment was done. Radiological assessment was ordered when indicated, especially in patients with intermediate or high malignant-risk potential.

The surgical specimen was sent to the histopathology laboratory for analysis. Immunohistochemistry was performed to distinguish these tumors from other subepithelial GIT tumors, and these included CD117, DOG-1, CD34, S-100, and smooth muscle actin (SMA). Mitotic activity, mitotic index, and malignant potential were also assessed and recorded. Assessment of the latter item was based on tumor size (> 5 cm) and mitotic count (> 10/10 high power field).

Data analysis was performed by Statistical Package for the Social Sciences (SPSS 26.0, IBM/SPSS Inc., Chicago, IL) software for Mac. Categorical data were expressed as frequencies and percentages (%), while in the quantitative data, we used mean and standard deviations (SD) as well as median (range).

## Results

Thirty-four patients underwent surgical management for small bowel GIST in the study duration at Gastrointestinal Surgical Center, Mansoura University. The mean age at presentation was 58.15 years (± 12.65), with a male to female ratio of 1.3:1.Reviewer (2) The most common presentation was abdominal mass (64.7%) followed by melena (52.9%). Patients’ demographic data and baseline characteristics are summarized in Table 1.

**Table 1:** Demographic data and duration of symptoms

	Total number=34		
	mean ± SD	Median	Range
**Age/years**	58.15 ± 12.65	57	(29-79)
**Sex**			
Males	19 (55.9%)		
Females	15 (44.1%)		
**Duration of symptom (Years)**	4.62 ± 2.34	5	(1-9)
**Complaint**			
Pain	17 (50%)		
Mass	22 (64.7%)		
Melena	18 (52.9%)		
Weight loss	5 (14.7%)		
Jaundice	2 (5.9%)		

Upper GI endoscopy revealed no abnormality in 32 cases (94.1%) and non specific findings (antral gastritis) in 2 (5.9%). Reviewer (2). Colonoscopic examination Reviewer (2).was performed in 7 cases; colonic polyp was detected in one case, whereas the remaining six showed unremarkable findings.

**Fig. 1:**
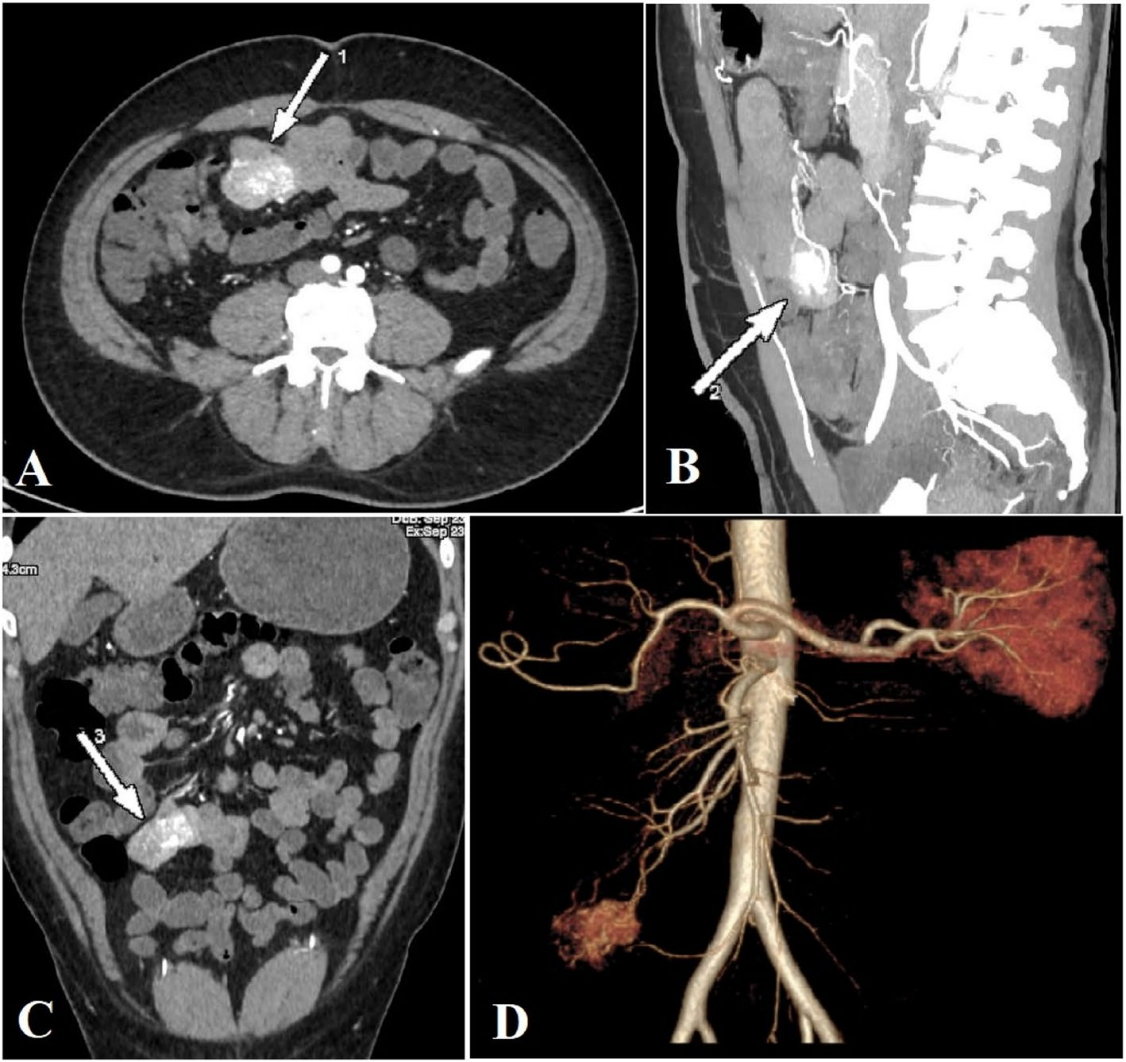
**A**, **B** and **C** Triphasic pelviabdominal cross sectional, sagittal and coronal CT angiogram Reviewer (2), views showing a hypervascular ileal GIST. **D** 3D reconstruction of the CT angiogram Reviewer (2), showing that the mass supplied from the ileocolic artery

Regarding radiological data, abdominal CT revealed a mass in 19 patients (55.9%), but the remaining cases showed no abnormalities in the same examination. Angiography was performed only in 15 cases (44.1%) as in Fig. 1. The previous data are summarized in Table 2.

**Table 2:** Endoscopic and radiological findings in the study sample

Variable	No (%)
**UGI Findings**	
No abnormality	32 (94.1%)
Antral gastritis	2 (5.9%)
**LGI Findings**	
Not done	27 (79.4%)
No abnormality	6 (17.6%)
Polyp	1 (2.9%)
**CT**	
No abnormality	15 (44.1%)
Mass	19 (55.9%)
**Angiography**	
Not done	19 (55.9%)
Done	15 (44.1%)

The mean lesional size was 8.76 cm (range 1.5—35.0 cm). Twenty lesions (58.8%) were found in the ileal region and the remainder in the jejunal region. Reviewer (2).

Pancreatic infiltration was detected in one case (2.9%), and another case had colonic involvement (2.9%).

**Fig. 2:**
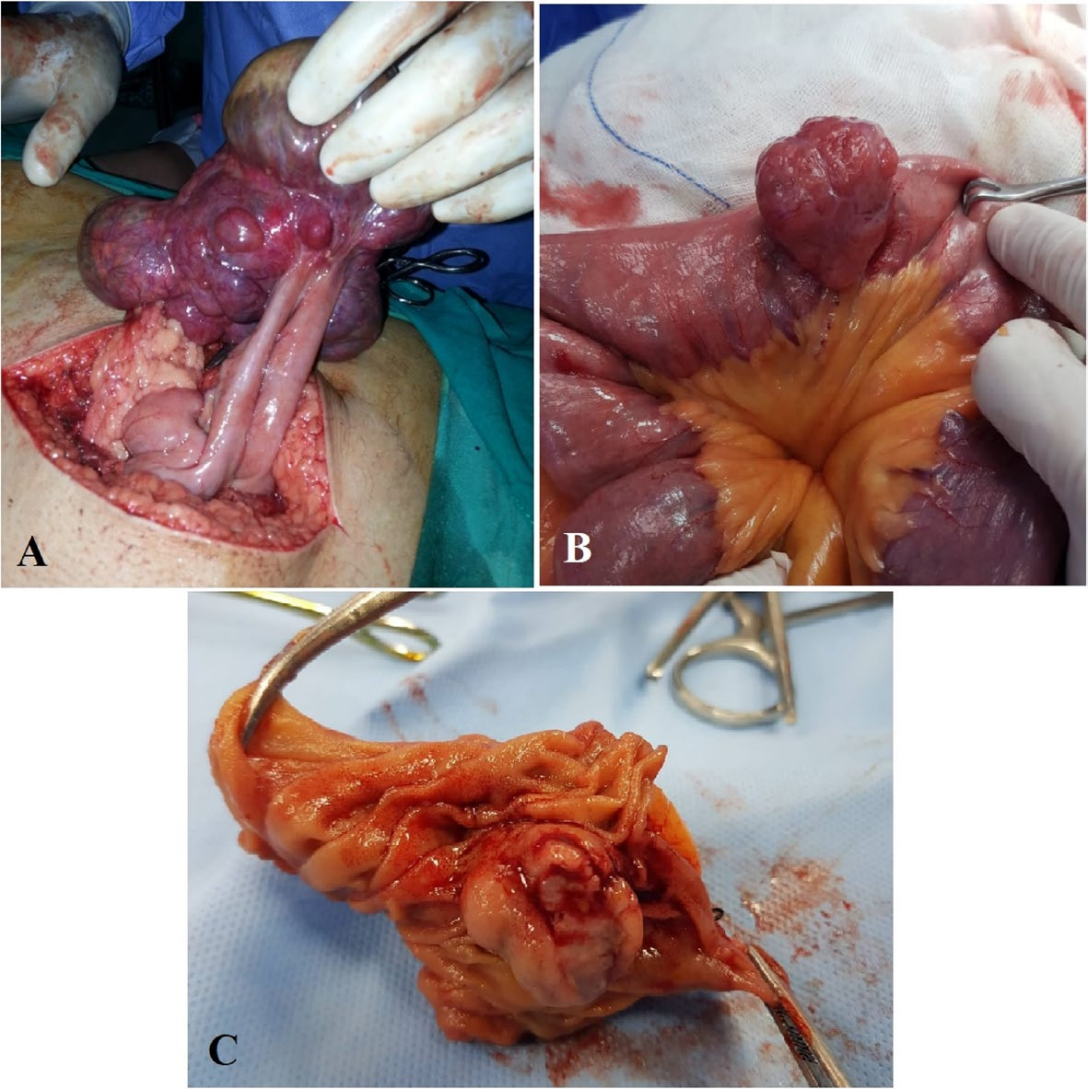
**A** Intraoperaive photo showing a large GIST arising from the small bowel wall. **B** Intraoperative photo showing a small GIST. **C** After resection and opening of the specimen of the same patient in (**B**), showing intraluminal ulcer

Surgery was the standard line of treatment. Resection anastomosis of the bowel with adequate safety margin was performed in all cases with en-bloc resection of involved organs as in Fig. 2. Table 3 summarizes operative findings in the study population.

**Table 3:** Operative data

Variable	Mean, SD, and range/No (%)		
**Mass size (cm)**	8.76 ± 7.76	6	(1.5-35)
**Site**			
Jejunum	14 (41.2%)		
Ileum	20 (58.8%)		
**Surrounding soft tissue infiltration**			
Pancreas	1 (2.9%)		
Colon	1 (2.9%)		

**Fig. 3:**
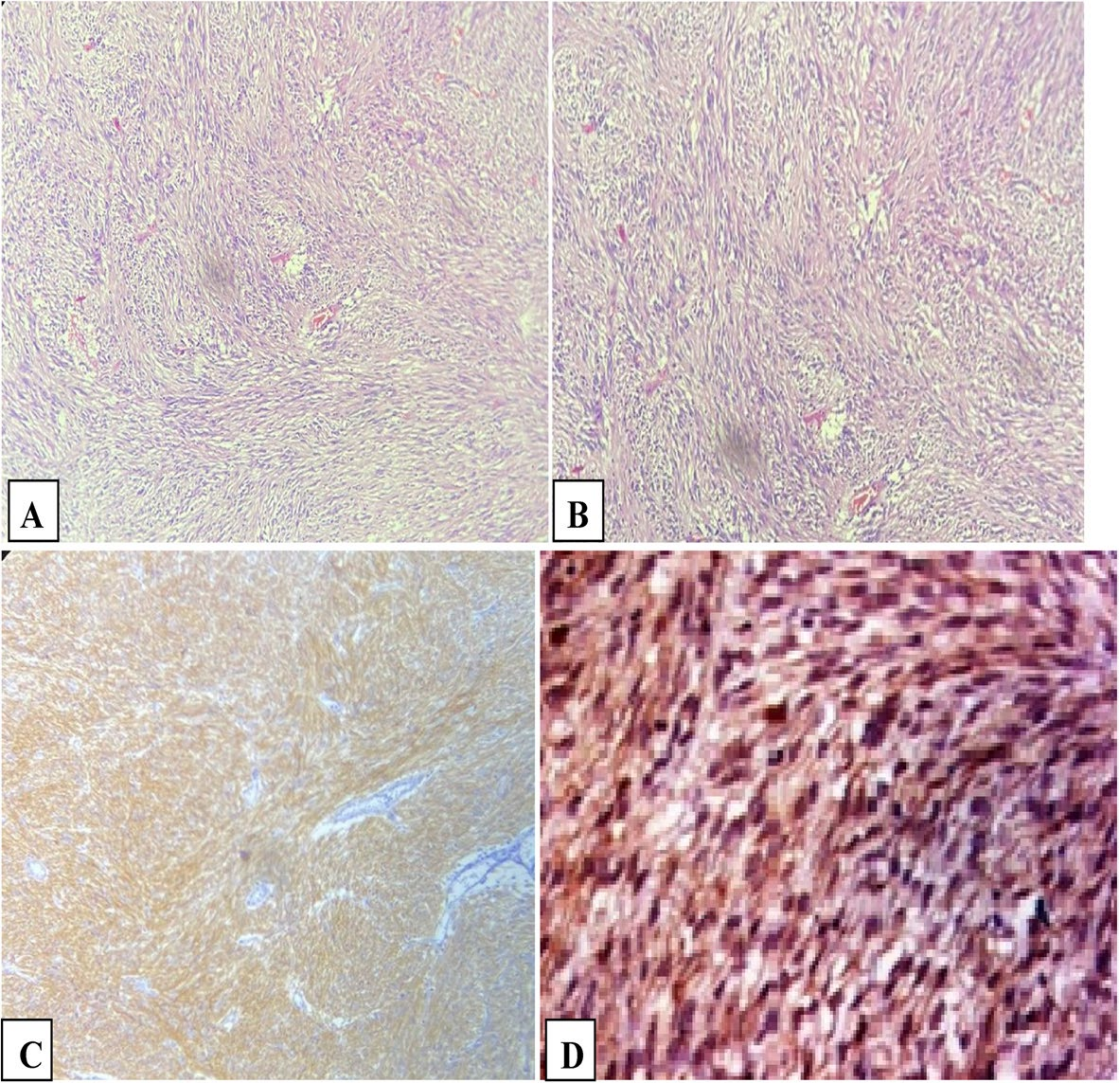
Histomicrographs of GIST. **A**, **B** The tumor is formed of spindle cell proliferation in fasicular pattern with oval shaped nuclei (H&E × 200). **C**, **D** The neoplastic cells showed positive cytoplasmic staining for CD117 (IHCx200). Reviewer 1

After pathological analysis. Fig. 3. reviewer (1), of the excised surgical specimen, 17 patients had GISTs with low malignant potential (50%), while 11 patients had high-malignant-potential lesions (32.4%). The remaining patients had moderate potential lesions.

Mitotic figures were absent in five cases (14.7%). However, we detected < 5/50 mitotic figures in 17 patients (50%) whereas > 5/50 mitotic figures were detected in ten patients (29.4%).IHC assessment revealed positivity for the used stains as follows; CD 117 (97.1%), DOG-1 (44.1%), CD 34 (76.5%), S-100 (8.8%) and SMA (8.8%). The surgical cut margin examination revealed its infiltration in two cases (5.9%), while the remaining cases had a free cut margin. Table 4 shows these data.

**Table 4:** Pathological data

Variable	No (%)
**Malignant potential**	
Low	17 (50%)
Moderate	6 (17.6%)
High	11 (32.4%)
**Mitotic figures**	
Not detected	5 (14.7%)
< 5/50	17 (50%)
> 5/50	10 (29.4%)
5/10	1 (2.9%)
<150	1 (2.9%)
**IHC**	
CD 117	33 (97.1%)
DOG-1	15 (44.1%)
CD 34	26 (76.5%)
S-100	3 (8.8%)
SMA	3 (8.8%)

During the scheduled follow-up period, recurrence was detected in only one patient (2.9%). Recurrence occurred in the small intestine and was managed by resection anastomosis. No mortality was encountered in the current study, as shown in Table 5.

**Table 5:** Outcomes in the study sample

Outcome	No (%)
Recurrence	1 (2.9%)
Mortality	0 (0%)

## Discussion

Diagnosis and management of small bowel GIST is a clinical challenge. A high grade of suspicion is required for early diagnosis. Thirty-four patients were included in the current study. The mean age of the study population was 58.15 (± 12.65) years. Zhou et al., in their retrospective series, which included 32 patients, their median age was 56 years and ranged between 23 and 81 years [18]. Another study included 197 patients; their ages ranged between 17 and 82 years, with a mean age of 53.97 years [17].

In the current study, male patients constituted 55.9% (*n* = 19) of the study population. Ost of the available studies reported a slightly higher predominance of the male sex Reviewer (2). [17, 24, 25]. While other studies reported no gender predominance in the literature [18, 26].

There is usually a lag between the onset of symptoms and diagnosis of GIST. In the current study, the mean duration of symptoms was 4.62 years ranging from one to nine years. Many causes could explain the long duration of symptoms.

First of all, the symptoms are non-specific and are similar to the manifestations of many abdominal pathologies. The GIST lesions usually grow slowly [17]. Additionally, the nature of the mass could not be precisely determined with radiological imaging alone [18]. Also, endoscopic access to the small bowel is not present in many centers. Yan and his associates confirmed our findings regarding the long duration of symptoms. The duration of manifestations had mean values of 52.6 and 77.9 months in the high and low-grade small bowel GISTs, respectively [27].

In the current study, the presence of a mass was the commonest Reviewer (2) (64.7%), followed by melena (52.9%) and abdominal pain (50%). Other symptoms included weight loss (14.7%) and jaundice (5.9%). Another study reported that GIT bleeding was the commonest symptom (46%), abdominal pain (18.8%), mass (12.5%), abdominal distension (9.4%), and anemia (3.1%). Three lesions were discovered incidentally Reviewer (2) [18].

Another study even stated that the presenting symptom would differ according to the location of the lesion. Authors reported that luminal bleeding was the commonest presentation for duodenal lesions (25%), while epigastric symptoms were more common with jejunal and ileal lesions (43.9% and 38.3%, respectively) [17].

There was no diagnostic value of endoscopic examination except in exclusion of gastric, duodenal, or colonic etiology attributable to the symptoms. Zhou et al. negated the identification of any hemorrhagic pathology in their cases diagnosed with small bowel GIST, who underwent both endoscopies [18].Both upper and lower GI endoscopies are of great value in GIST lesions. Even if they cannot detect lesions, they will exclude other differential causes of patient symptoms [28]. When using endoscopic ultrasound, it adds a great advantage, as it offers a wide range of view, especially if it is related to the gastric or duodenal walls, and an endoscopic biopsy could be obtained to confirm the disease [28, 29]. Also it should be mensioned, EUS-FNA is considered as a well tolerated and feasible endoscopic microsurgery to confirm a diagnosis of a suspected GIST [30]. Reviewer (1).

In the current study, CT detected a mass in 19 patients (55.9%). Mesenteric angiography was diagnostic in the remaining cases and revealed an intestinal vascular lesion. In agreement with the previous findings; two previous studies have confirmed the efficacy and feasibility of CT angiography in diagnosing such lesions, with a sensitivity of 90.9% for small bowel GIST [31, 32]. In a similar study, CT provided a provisional diagnosis of small intestinal GIST in 17/32 patients (54.8%). In the same study, angiography revealed an intestinal vascular lesion in 5/7 patients (71.4%) [18].

Our findings showed that 14 patients had jejunal lesions (41.2%), whereas the remaining patients (58.8%) had ileal lesions. On the other hand, in the study conducted by Baheti and his associates, 64 patients (62.75%) had jejunal tumors, while 38 patients had ileal lesions (37.25%)[25].Other reported the site of the include lesions as follows; 19 lesions in the duodenum, 63 in the jejunum, and 17 in the ileum [24].The previous authors reported a higher prevalence of jejunal lesions.The difference in sample size between different studies could explain these differences.

In the current study, Lesion size had a mean value of 8.76 cm, and it ranged between 1.5 and 35 cm. Baheti et al. reported that the tumors of the included 102 patients with small bowel GISTs had a mean size of 8.5 cm (range, 2 – 28 cm) [25]. Another series, including 27 small intestinal GIST lesions, reported that the mean size was 8.5 cm [33]. Giuliano et al. reported that tumor size had a median size of 6.2 cm (IQR 3.8 – 10 cm) [34]. These authors supported that the mean GIST size gradually increased from the duodenum to the ileum, which explains our large size range, as most of our cases had ileal lesions.

In the current study, small intestinal resection-anastomosis was performed for all patients. The two cases with surrounding organ infiltration (colon and pancreas) were managed by right hemicolectomy and distal pancreatectomy in the same session. Currently, radical surgical resection is the gold standard option for small bowel GISTs [35]. The adequacy of radical resection is assessed could be assessed by borderline status along with complete resection without tumor overflow or rupture [36, 37]. A previous study reported that segmental intestinal resection was performed for 27 patients (84.4%) with contiguous organs involved (in 9 cases), pancreaticoduodenectomy for two cases with duodenal lesions (6.2%), and local excision in three patients with isolated tumors [18].

Our findings showed that CD 117 was positive in the majority of patients (97.1%). It is agreed that CD 117 (KIT) is the most prominent diagnostic marker for GIST, with about 95% of lesions are positive for CD 117 on IHC [38–40]. However, some GIST lesions that show positivity for KIT are negative for its mutations. These tumors will stain positive for KIT with no detectable mutations on the gene itself. These patients are expected to have a poor response to imatinib therapy [41]. In our study, the resected GIST lesions showed 76.5% positivity for CD 34 (76.5%).

Likewise, Hirota et al. reported that about 72 – 78% of GIST lesions were positive for CD 34 [42]. We detected DOG-1 in 15 out of the included 34 patients (44.1% positivity rate). However, multiple previous studies reported higher positivity rates for the same marker, which was 97.8% as reported by West et al. [43] and 87% by Sözütek et al. [44].

Our findings showed that S-100 and SMA showed positivity in 8.8% of patients (three cases for each marker). Other previous studies stated that between 30 to 40% of GIST show positivity for SMA, whereas only 5% show positivity for S-100 protein [42, 45, 46].

When it comes to pathological classification, half of our cases were low grade, while high-grade lesions were detected in 32.4% of patients. The remaining patients (17.6%) had intermediate lesions. Beheshti et al. reported the low-, intermediate- and high- malignant risk small bowel GISTS were detected in 20.59%, 16.67%, and 62.64% of patients, respectively [25].

It is expected to find some differences between various studies regarding the malignant potential of the included tumors due to different tumor criteria, grading methods, or sample size included. The difference in tumour location could be another explanation, as previous authors reported a higher prevalence of high-risk lesions in the ileum compared to the proximal small bowel portions [17].

We did not encounter any cases of mortality in the scheduled follow-up period. Also, recurrence was detected in only one case (2.9%). Zhou and his colleagues reported no recurrence during the planned 30-month follow-up period (range, 3 – 54) [18].

Other authors reported a higher recurrence rate, as 44 out of 85 patients who underwent curative resection developed recurrence. Recurrence occurred within 3.7 – 125.1 months after the operation. Disease-free survival was 85.2%, 53.8%, and 43.7% after 1, 3, and 5 years, respectively [24].

One could see differences in postoperative recurrence rates throughout the literature, and different tumor characteristics could explain this, the operation performed postoperative adjuvant regimen, or follow-up duration.

The current study has some limitations. Small sample size, along with its retrospective nature, are the main cons. Hence, an international registry might be useful Reviewer (2), including more cases from different surgical centers, especially with this rare clinical entity.

## Conclusion

Small bowel GISTs often need a long time to be diagnosed. Implementing new diagnostic techniques like angiography, capsule endoscopy, and enteroscopy should be encouraged when suspecting these lesions. Surgical resection is always associated with an excellent postoperative recovery profile and very low recurrence rates.

## Data Availability

The data that support the findings of this study are available from the corresponding author, upon reasonable request.
